# Supplementary material for: Long-lasting field-free alignment of large molecules inside helium nanodroplets
Source: Nat Commun. 2019 Jan 11;10:133. doi: 10.1038/s41467-018-07995-0 (PMC6329814; doi:10.1038/s41467-018-07995-0)
Supplement: Supplementary file 1 — Supplementary Information [file 41467_2018_7995_MOESM1_ESM.pdf]

Supplementary Information for ‘Long-lasting field-free alignment of large molecules inside helium nanodroplets’

Chatterley et al.

Supplementary Note 1: Demonstration of 3D alignment

In the main text, both DBT and TBI were 3D aligned using an elliptically polarized laser pulse (3:1 intensity ratio). An elliptically polarized pulse constrains all three axes of the molecule in the laboratory frame, as opposed to a linearly polarized pulse, which only confines the most polarizable axis (1D alignment) and leaves the molecule free to rotate around this axis.

5,5′′-Dibromo-2,2′:5′,2′′-terthiophene (DBT)

Coulomb explosion of DBT produces both Br^+ and S^+ ions, which depart with (approximately) perpendicular velocities. In an identical manner to our previous work on 3,5-dichloroiodobenzene[1] we can thus use the Br^+ ions in the side view and S^+ ions in the end view to describe the 3D alignment. (See Fig. 2 in the main text for an explanation of 'end' and 'side' views). Supplementary figure 1 shows the Br^+ and S^+ ion images at the peak of the alignment pulse. With the linearly polarized alignment pulse, the Br^+ ions are strongly confined along the polarization vector (the Y-axis) showing that the most polarizable axis (MPA) is aligned along this axis. The simultaneous observation of a circularly symmetric S^+ ion image shows that the second most polarizable axis (SMPA) is randomly distributed, that is the molecule is free to rotate around its MPA. This is the expected behavior for 1D alignment.[2]

When the elliptically polarized pulse is used, the Br^+ ions remain confined along the Y-axis. The circular symmetry of the S^+ ions is, however, broken and confinement of this ion species along the Y-axis is observed. This implies that the SMPA is also aligned and thus we conclude, in line with previous works, that the DBT molecules are 3D aligned.

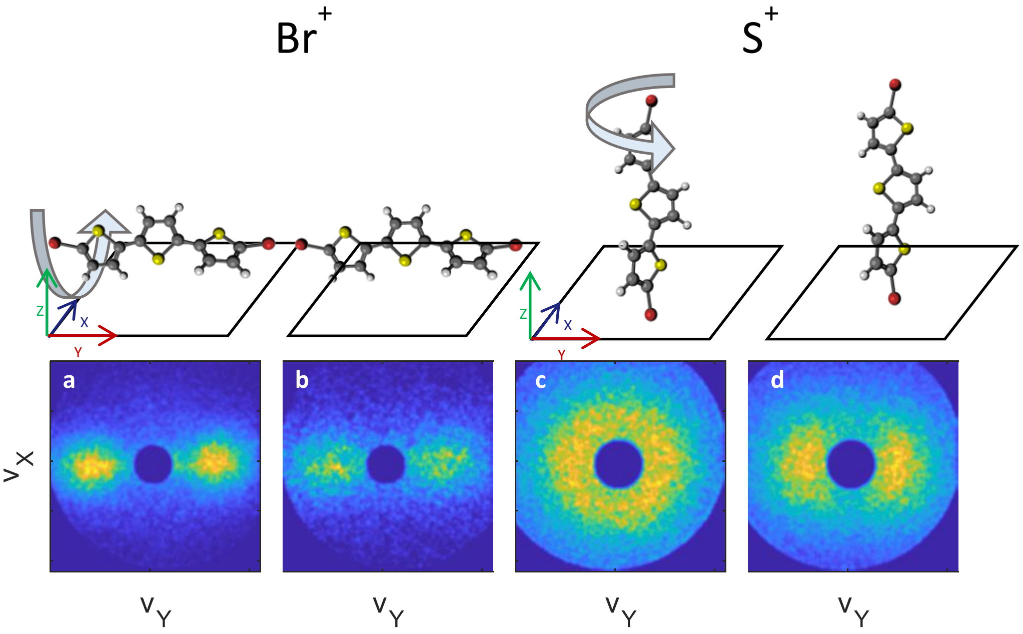

Supplementary figure 1: Demonstration of 3D alignment in DBT. Ion images of Br^+ (**a,b**) and S^+ (**c,d**) produced from Coulomb explosion of DBT molecules at the peak of either a linearly polarized (**a,c**) or an elliptically polarized (**b,d**) alignment pulse with a peak intensity of 9×10^{11} W / cm^2.

5,7,5',7'-Tetrabromoindigo (TBI)

In the experiments on the TBI molecule, we only recorded Br^+ ions, so an analysis similar to the one for DBT is not possible. However, by considering both ion images and the corresponding angular covariance maps,[3] we can definitively demonstrate that this molecule also 3D aligns.

Supplementary figure 2 shows Br^+ images, recorded in the side view, for a linearly polarized (**a**) and an elliptically polarized (**b**) alignment pulse, as well as the corresponding angular covariance maps (**c** and **d**). The covariance maps reveal possible correlations in the emission direction of the Br^+ ions from Coulomb explosion and thus can be used to identify which positions on the molecule the Br^+ ions originate from.

For a linearly polarized alignment pulse the Br^+ image shows two pronounced blobs, marked by the purple circles, confined along the Y-axis. We interpret these blobs as the result of the Br^+ ions originating from the Br atoms in the 5 and 5' position on the molecule, and so from the end of the molecule (see labelling in supplementary figure 2). A linearly polarized pulse should align the MPA along its polarization (coinciding with the Y-axis). Since the axis connecting the 5-Br and the 5'-Br lies close to the MPA it should also be confined along the Y-axis and thus the corresponding Br^+ ions should recoil approximately on this axis, consistent with the observation. The angular covariance map corroborates this interpretation by revealing a strong correlation between Br^+ ions ejected at θ = 0° and θ = 180°, where θ is the angle between the emission direction of the Br^+ ion and the Y-axis.

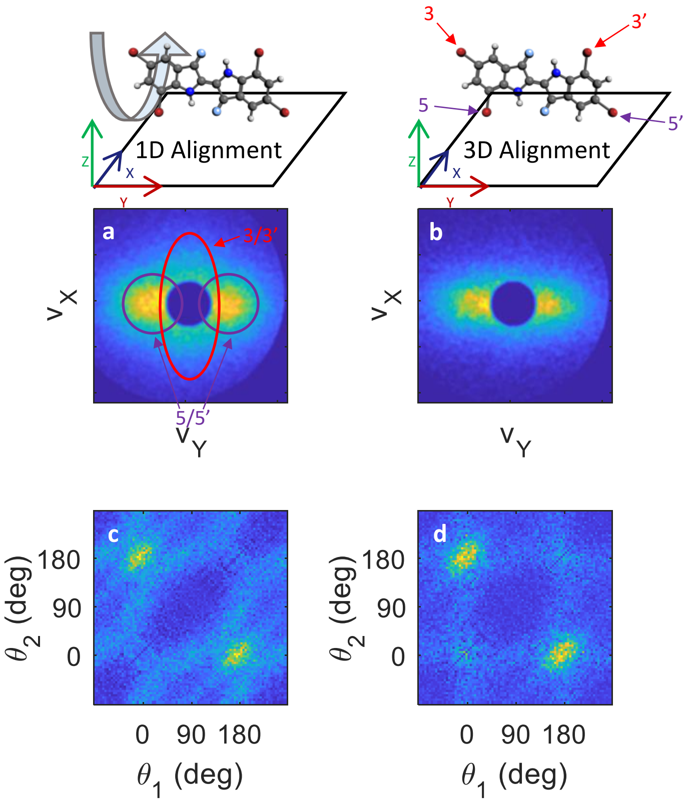

Supplementary figure 2: Demonstration of 3D alignment in TBI. Ion images (**a,b**) and angular covariance maps (**c,d**) of TBI at the peak of alignment in side view, with either 1D (**a,c**) or 3D (**b,d**) alignment. In the cartoons at the top, the detector is positioned below the molecules and the most polarizable axis is parallel to the Y axis. In **a**, the position of the 3/3' and 5/5' Br^+ ions are indicated on the image.

The Br^+ ion image also shows a signal, marked by the red ellipse, confined along the X-axis. We interpret this signal as stemming from the Br^+ ions originating from the 7 and 7´ positions on the molecule. The linearly polarized alignment pulse should only align the MPA but will leave the molecule free to rotate around this axis. Therefore, the Br^+ ions from the 7 and 7´positions should be ejected in a ring normal to the MPA, which upon projection on the detector should result in a stripe. This is consistent with the signal observed inside the red ellipse on supplementary figure 2**a**. Again, the angular covariance map corroborates this interpretation: delocalized diagonal stripes with a relative angle of 90° are visible, indicating that 7 or 7' Br^+ ions are detected in coincidence with an aligned 5 or 5' Br^+ ion, with all starting angles equally likely.

For the elliptically polarized pulse, the confined blobs along the Y-axis are still present showing that that the MPA remains aligned. The signal along the X-axis has, however, disappeared. This shows that the 7 or 7' Br^+ ions are no longer randomly ejected around the Y-axis. Instead, they must be ejected perpendicular to the detector plane, and so along the Z-axis. Since the emission direction of the 7 or 7' Br^+ ion should approximately indicate the

alignment of the SMPA we conclude that the SMPA is aligned along the Z-axis, and so the molecule is 3D aligned. This is corroborated by the vanishing of the diagonal stripes in the covariance map, indicating that we can no longer detect coincident ion pairs from the lateral (7,7') or end (5,5') positions of the Br atoms.

Supplementary Note 2: Simulations of gas phase I_2

To rule out the possibility that the long-lived field-free alignment we observe in helium droplets can be explained as either an effect of temperature or modified moment of inertia, we have performed simulations of the alignment dynamics of gas phase I_2. The simulations were performed by solving the time dependent Schrodinger equation for a linear I_2 molecule in the presence of an electric field with an envelope and intensity matching the experimental pulse. The initial state population was determined from a Boltzmann distribution, and focal volume averaging effects were taken into account. Details on the simulations have been presented previously.[4,5]

Supplementary figure 3 presents the results of these simulations, along with the experimental alignment dynamics for I_2 in both the gas phase and solvated in a helium droplet. As a benchmark, dynamics were simulated for I_2 at the estimated experimental gas phase rotational temperature of 1 K (blue line). The alignment minimum is at 14 ps, in good agreement with the experimental minimum at 13 ps (dotted line). Next, we simulated the gas phase dynamics at 0.4 K, the temperature inside helium droplets (orange line). The minimum shifts to 18 ps, however this is still very far from the experimental helium solvated minimum at 35 ps, hence temperature alone cannot explain the slower dynamics in helium droplets.

Secondly, we considered the possibility that slow dynamics in helium droplets are due to an increase of the effective moment of inertia. Rotational spectroscopy has shown that helium solvation increases the effective moment of inertia for many molecules I_{eff} by as much as around three-fold.[6,7] The effective moment of inertia in I_2 has been estimated as $I_{\mathrm{eff}} = 1.7 \times I_0$, where I_0 is the moment of inertia for isolated I_2.[8] We see that neither 1.7 nor $3 \times I_0$ (yellow & purple lines) are sufficient to reproduce the experimental minimum. In fact, an $I_{\mathrm{eff}} \approx I_0 \times 10$ is required to sufficiently delay the alignment minimum. Such a large I_{eff} is unphysical, and hence we can also rule out simple effective moment of inertia increases as the cause of the slow dynamics.

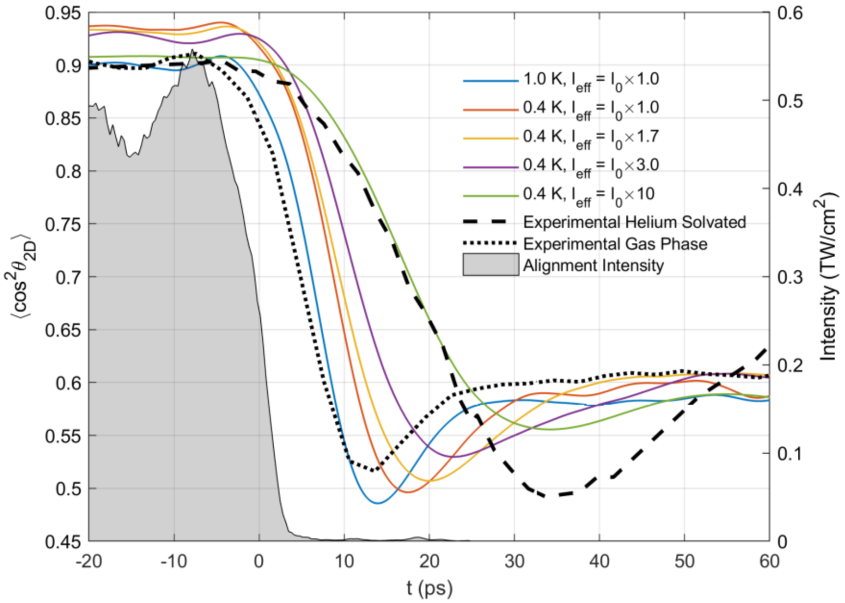

Supplementary figure 3: The early time alignment dynamics of I_2 with truncated pulses. Black dashed and dotted lines, respectively, show experimental values in helium droplet and gas phase environments. Coloured lines show simulated gas phase dynamics for different rotational temperatures and effective moments of inertia.

Supplementary Table 1: Molecular Polarizabilities and Moments of Inertia

Supplementary table 1 lists the polarizability and moment of inertia tensors for the molecules used in this study. The reference frame is chosen such that the polarizability tensor is diagonal. The molecular structures are shown with the most polarizable axis horizontal, the second most polarizable axis vertical, and the third most polarizable axis going into the page; the coordinate system used is defined in the top-left cell. The arrows under the molecular structures show the A and B axes of rotation in this frame. The moment of inertia tensors are shown in the same polarizability reference frame. Off-diagonal elements are seen for DBTP and TBI, indicating that the axes of most polarizability and inertia do not coincide exactly. The values for I_2 were taken from the literature,[9] while the other molecules were calculated using a density functional method ,Wb97xD,[10] in the Gaussian09 package.[11] For DBTP and TBI, the basis set was aug-pcseg-n from Jensen and coworkers[12] while for DIBP Def2QZVPP[13] was chosen due to the presence of the iodine atom.

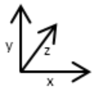	$\begin{pmatrix} \alpha_{xx} & \alpha_{xy} & \alpha_{xz} \\ \alpha_{yx} & \alpha_{yy} & \alpha_{yz} \\ \alpha_{zx} & \alpha_{zy} & \alpha_{zz} \end{pmatrix}$ (Å^3)	$\begin{pmatrix} I_{xx} & I_{xy} & I_{xz} \\ I_{yx} & I_{yy} & I_{yz} \\ I_{zx} & I_{zy} & I_{zz} \end{pmatrix}$ (amu Å^2)
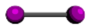	$\begin{pmatrix} 14.6 & 0 & 0 \\ 0 & 8.5 & 0 \\ 0 & 0 & 8.5 \end{pmatrix}$	$\begin{pmatrix} 0 & 0 & 0 \\ 0 & 451 & 0 \\ 0 & 0 & 451 \end{pmatrix}$
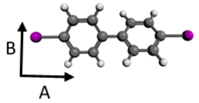	$\begin{pmatrix} 54 & 0 & 0 \\ 0 & 26 & 0 \\ 0 & 0 & 19 \end{pmatrix}$	$\begin{pmatrix} 177 & 0 & 0 \\ 0 & 8800 & 0 \\ 0 & 0 & 8921 \end{pmatrix}$
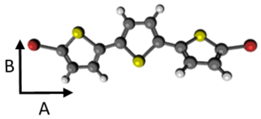	$\begin{pmatrix} 66 & 0 & 0 \\ 0 & 30 & 0 \\ 0 & 0 & 23 \end{pmatrix}$	$\begin{pmatrix} 304 & -0.04 & -121 \\ -0.04 & 10381 & 0 \\ -121 & 0 & 10534 \end{pmatrix}$
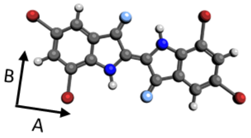	$\begin{pmatrix} 86 & 0 & 0 \\ 0 & 48 & 0 \\ 0 & 0 & 23 \end{pmatrix}$	$\begin{pmatrix} 2760 & -1583 & 0.04 \\ -1583 & 12738 & -0.04 \\ 0.04 & -0.04 & 15498 \end{pmatrix}$

Supplementary References

1 Chatterley, A. S., Shepperson, B. & Stapelfeldt, H. Three-Dimensional Molecular Alignment Inside Helium Nanodroplets. *Phys. Rev. Lett.* **119**, 073202, doi:10.1103/PhysRevLett.119.073202 (2017).

2 Stapelfeldt, H. & Seideman, T. Colloquium: Aligning molecules with strong laser pulses. *Rev. Mod. Phys.* **75**, 543-557, doi:10.1103/RevModPhys.75.543 (2003).

3 Hansen, J. L. *et al.* Control and femtosecond time-resolved imaging of torsion in a chiral molecule. *J. Chem. Phys.* **136**, 204310, doi:10.1063/1.4719816 (2012).

4 Chatterley, A. S. *et al.* Communication: Switched wave packets with spectrally truncated chirped pulses. *J. Chem. Phys.* **148**, 221105, doi:10.1063/1.5028359 (2018).

5 Søndergaard, A. A., Shepperson, B. & Stapelfeldt, H. Nonadiabatic laser-induced alignment of molecules: Reconstructing <cos2θ> directly from <cos2θ2D> by Fourier analysis. *J. Chem. Phys.* **147**, 013905, doi:10.1063/1.4975817 (2017).

6 Grebenev, S. *et al.* The rotational spectrum of single OCS molecules in liquid 4He droplets. *J. Chem. Phys.* **112**, 4485-4495, doi:10.1063/1.481011 (2000).

7 Toennies, J. P. & Vilesov Andrey, F. Superfluid Helium Droplets: A Uniquely Cold Nanomatrix for Molecules and Molecular Complexes. *Angew. Chem. Int. Ed.* **43**, 2622-2648, doi:10.1002/anie.200300611 (2004).

8 Shepperson, B. *et al.* Laser-Induced Rotation of Iodine Molecules in Helium Nanodroplets: Revivals and Breaking Free. *Phys. Rev. Lett.* **118**, 203203, doi:10.1103/PhysRevLett.118.203203 (2017).

9 Maroulis, G., Makris, C., Hohm, U. & Goebel, D. Electrooptical Properties and Molecular Polarization of Iodine, I2. *J. Phys. Chem. A* **101**, 953-956, doi:10.1021/jp962578u (1997).

10 Chai, J.-D. & Head-Gordon, M. Long-range corrected hybrid density functionals with damped atom–atom dispersion corrections. *Phys. Chem. Chem. Phys.* **10**, 6615-6620, doi:10.1039/B810189B (2008).

11 Gaussian 09, Revision B.01 (Wallingford CT, 2009).

12 Jensen, F. Unifying General and Segmented Contracted Basis Sets. Segmented Polarization Consistent Basis Sets. *Journal of Chemical Theory and Computation* **10**, 1074-1085, doi:10.1021/ct401026a (2014).

13 Weigend, F. Accurate Coulomb-fitting basis sets for H to Rn. *Phys. Chem. Chem. Phys.* **8**, 1057-1065, doi:10.1039/B515623H (2006).
